# NMDA Receptor Opening and Closing—Transitions of a Molecular Machine Revealed by Molecular Dynamics

**DOI:** 10.3390/biom9100546

**Published:** 2019-09-28

**Authors:** Jiří Černý, Paulína Božíková, Aleš Balík, Sérgio M. Marques, Ladislav Vyklický

**Affiliations:** 1 Institute of Physiology of the Czech Academy of Sciences, Vídeňská 1083, 142 20 Prague, Czech Republic; ales.balik@fgu.cas.cz; 2 Institute of Biotechnology of the Czech Academy of Sciences, BIOCEV, Průmyslová 595, 252 50 Vestec, Prague West, Czech Republic; Paulina.Bozikova@ibt.cas.cz; 3 Loschmidt Laboratories, Department of Experimental Biology and RECETOX, Masaryk University, Kamenice 5/A13, 625 00 Brno, Czech Republic; smar96@gmail.com; 4 International Centre for Clinical Research, St. Anne’s University Hospital Brno, Pekařská 53, 656 91 Brno, Czech Republic

**Keywords:** glutamate receptor gating, molecular modeling, molecular dynamics simulations, NMDA receptor transition, open and closed state

## Abstract

We report the first complete description of the molecular mechanisms behind the transition of the *N*-methyl-d-aspartate (NMDA) receptor from the state where the transmembrane domain (TMD) and the ion channel are in the open configuration to the relaxed unliganded state where the channel is closed. Using an aggregate of nearly 1 µs of unbiased all-atom implicit membrane and solvent molecular dynamics (MD) simulations we identified distinct structural states of the NMDA receptor and revealed functionally important residues (GluN1/Glu522, GluN1/Arg695, and GluN2B/Asp786). The role of the “clamshell” motion of the ligand binding domain (LBD) lobes in the structural transition is supplemented by the observed structural similarity at the level of protein domains during the structural transition, combined with the overall large rearrangement necessary for the opening and closing of the receptor. The activated and open states of the receptor are structurally similar to the liganded crystal structure, while in the unliganded receptor the extracellular domains perform rearrangements leading to a clockwise rotation of up to 45 degrees around the longitudinal axis of the receptor, which closes the ion channel. The ligand-induced rotation of extracellular domains transferred by LBD–TMD linkers to the membrane-anchored ion channel is responsible for the opening and closing of the transmembrane ion channel, revealing the properties of NMDA receptor as a finely tuned molecular machine.

## 1. Introduction

Early pharmacological experiments have suggested the existence of several distinct excitatory amino acid receptors that may be distinguished by the use of selective agonists and antagonists with *N*-methyl-d-aspartic acid (NMDA) being a specific agonist for one type of receptor [1]. Later, these results were confirmed by molecular cloning and it was shown that NMDA receptors (NMDARs) are hetero-tetramers, composed of four subunits—two GluN1 and two GluN2A-D and/or GluN3A-B subunits [2,3,4,5,6]. It is now well established that NMDARs play a crucial role in rapid excitatory synaptic transmission in the mammalian central nervous system, they promote persistent changes in synaptic strength [7,8,9], and states associated with excessive receptor activation, as well as receptor hypo- or hyper-function are clinically relevant [10,11,12,13].

Glutamate and glycine binding to the respective ligand-binding domains at the GluN2 and GluN1 subunit promote conformational changes leading to the opening of the channel pore that is permeable to sodium and calcium [14]. Kinetic analysis of macroscopic responses induced by glutamate in the presence of a saturating concentration of glycine reveal a minimum of five states that describe receptor agonist activation, desensitization, and channel opening [15,16]. If we take into account glycine binding [17,18] and calcium-induced inactivation [19,20] 12 states of the NMDAR can be predicted. Single-channel analysis shows additional states for agonist-bound receptors, which includes a minimum of five closed states and at least two open states [21,22,23,24,25,26].

Recent crystallographic data represent significant progress in understanding the molecular organization of the NMDAR [27,28], however, the precise molecular details of the dynamic processes associated with receptor transition from the closed to open conformation are unknown. Existing crystal structures allow identification of the most constricted region between the channel-lining M3 helices of the transmembrane domain (TMD). This gate, further referred to as TTTT due to the involved residues, is formed by the Thr648 residue of the GluN1 subunit and the Thr647 residues of the GluN2B subunit. Our recent combined experimental and theoretical study [29] also revealed the existence of an auxiliary gate formed by Leu657 of GluN1 and Ile655 of GluN2B, referred to as the LILI gate. There are several limits to the resolved structure of the NMDAR that preclude its association with any of the conformational states identified electrophysiologically. First, the receptor is crystallized with a truncated C-terminal domain (CTD), putative glycosylation sites removed, certain cysteine residues mutated to serine, new cysteine residues introduced, several residues completely removed, and some charged residues neutralized [27], therefore it is quite likely that it reflects the structure of a non-functional receptor. Second, rapid transitions in between numerous conformational states predicted by electrophysiological/functional studies in a millisecond range preclude detailed structural analysis of the receptor.

Molecular simulation has proven useful in describing the dynamics and energetics of ligand binding to the NMDAR [30,31,32], receptor dynamics and channel opening [29,33,34,35,36,37,38], drug action [39,40], role of glycans [41,42] and conformational changes underlying disease-associated mutations in genes encoding NMDAR subunits [43,44,45]. This work concentrated on processes associated with receptor transition from the closed to open conformation, where the so far unknown closed conformation should correspond to the receptor not just when the ion channel is impermeable, but also structurally relaxed as a consequence of the glycine and glutamate ligands not bound nor acting on the receptor.

## 2. Materials and Methods

As described earlier [29], an all-atom model of the rat GluN1/GluN2B containing the extracellular and transmembrane parts (GluN1, UniProt [46] P35439, residues 23–847; GluN2B, UniProt Q00960, residues 30–852) was built with MODELLER version 9.14 [47] using the available crystal structures (4pe5 [27], 4tll [28], 4tlm [28]) as templates. Sequence alignment of the template sequences is available as a part of the Supplementary Material. The available crystal structures contain various unresolved regions without reliable structural information. For the simulations such regions missing in the template were modeled and further refined using the refine.fast routine of the loopmodel function of MODELLER, taking the lowest energy conformation of each patch of residues according to the discrete optimized protein energy (DOPE) score. This was important especially for the mostly unresolved region of linkers connecting ligand-binding domains (LBDs) and TMDs where the lowest energy conformations were selected from 1000 loopmodel refined models for each missing linker region. The remaining sequence differences in template structures introduced for crystallization purposes were also reverted to the wild-type reference sequences (see the SEQADV records in template PDB structures for a detailed list of differences and the Figure 3 of Reference [27] for their location within the structure). The most significant example was the GluN2B S214C amino-terminal domain (ATD) variant forming a disulphide bridge connecting both N2B subunits. This allowed crystallization by reducing receptor flexibility, but also enforced a close proximity of domains and rendered the receptor inactive or with only very low conductance.

The resulting GluN1/GluN2B homology model was further used for molecular dynamics simulations. The parameters of implicit solvation/lipid membrane model (EEF1/IMM1 [48]) were assigned using the web-based graphical user interface CHARMM-GUI [49]. The disulfide bridges were assigned manually according to the structural and UniProt data. The implicit solvent/membrane parameters were selected as follows: the hydrophobic thickness of the membrane was set to 26 Å, the anionic lipids fraction to 5%, salt concentration to 100 mM, and transmembrane voltage to 100 mV (positive in the extracellular part direction). The Langevin molecular dynamics simulation was then performed using the CHARMM [50] molecular dynamics package version c41b1, employing the topology and parameter definitions version 19. We introduced distance restraints between glycine and residue GluN1(R524) and glutamate and GluN2B(R519) for the liganded open-state simulation in order to compensate for their relatively low residence time resulting from the implicit solvation model. The simulation temperature was set to 298 K, the time step to 2 fs, and snapshots of geometry were collected every 10 ps for a simulation time of 150 ns for each simulation. In total, three simulations of unliganded receptor (closing simulations), three simulations of liganded receptor without ligand restraints, and three with ligand restraints (opening simulations) were performed. However, parts of the non-restrained simulations were discarded in the case of ligand unbinding. This led to nearly 1μs of molecular dynamics (MD) data composed of 3 × 150 ns of the unliganded simulation, 3 × 150 ns of the liganded simulation with ligand restraints, and portions of liganded unrestrained simulation data of varying size, where all ligands were bound in their LBDs.

The explicit solvation/membrane molecular dynamics simulation for the extracted GluN1/GluN2B transmembrane domain inserted in a 1,2-Dioleoyl-sn-Glycero-3-Phosphocholine (DOPC) membrane was performed using GROMACS [51] version 5.0.2 with the charmm36 force field. For the GluN1 TMD we used residues 540–674 and 789–843. For the GluN2B TMD we used residues 535–676 and 792–845. The system was prepared using the CHARMM-GUI web interface by placing the protein and membrane into a 159 × 159 × 129 Å simulation box filled with 100 mM NaCl solution in tip3p water. The system was equilibrated according to the standard protocol and the production 100 ns NPT simulation was performed using the Nosé–Hoover thermostat and the Parrinello–Rahman barostat. The geometry was collected every 10 ps. The graphical representation and geometry analysis of modeling results was performed using PyMOL version 2.2.0 [52] and VMD 1.8.7 [53]. Protein sequences were analyzed using the Unipro UGENE software version 1.31.0 [54].

The Markov states modeling (MSM) was performed using the HTMD package [55,56]. All the above-mentioned parts of trajectories for the opening and closing simulations, including their initial parts describing the relaxation of crystal-based model, were pooled and analyzed. The Markov states were identified based on the all C_α_ RMSD metric (without an a priori selection of “important” residues). The trajectories were clustered according to the RMSD parameter and six states were created using a 0.1 ns lag-time (based on the implied timescale plot). The memory intensive MSM analysis was performed on a 40 core, 1 TB RAM machine. The Markovian behavior of the constructed states was assessed using the Chapman–Kolmogorov test. The equilibrium population of each state was analyzed, and the flux rates were predicted. Each model was saved as an ensemble of 50 randomly selected representative structures and further compared to the manually obtained representative structures using PyMOL.

## 3. Results

Here, using an aggregate of nearly 1 µs of unbiased all-atom MD simulations in an implicit membrane and solvent, we investigated the molecular mechanisms of the receptor transition from the state with the transmembrane domain and the ion channel in the open configuration (with both glutamate and glycine bound to the respective sites at the GluN2B and GluN1 subunits) to the relaxed unliganded state with the channel closed.

Our NMDAR model covered the extracellular ATD, LBD, and transmembrane domain (TMD) of rat GluN1 and GluN2B. The structure of an intracellular CTD was not available in the current structural data and therefore CTDs were not included in the model. It was shown experimentally that the CTDs modulate ion channel conductance and gating [57], but these domains are not essential for correct function of the NMDAR. The native NMDAR is highly glycosylated and it was shown by experimental as well as theoretical studies that the glycans are essential for correct trafficking of the mature receptor to the cell membrane [58] and modulation of the affinity and kinetics of interactions between LBDs and their ligands [42]. On the other hand, missing glycosylation does not lead to a loss of NMDAR gating function. Supported by the available data, our model consisting of a crystal structure-based CTD-shortened receptor with non-glycosylated ATDs, LBDs and TMDs, should still have provided a reliable mechanistic description of NMDAR transitions between closed and open conformations.

Models of the open and closed states of the GluN1/GluN2B receptor were obtained from the MD simulation of the GluN1/GluN2B homology model with and without the glycine and glutamate ligands bound within the ligand-binding domains, respectively. To collect the simulation data for the opening of the GluN1/GluN2B receptor continuously influenced by the glycine and glutamate ligands we introduced distance restraints between glycine and residue GluN1(R524) and glutamate and GluN2B(R519).

### 3.1. Relaxation of the Crystal-Based Homology Model (NMDAR in the Agonist Activated Receptor State)

As already stated above, it was difficult to assign a functional state to the various NMDAR structures captured by crystallography or cryoEM experiments. Based on various distance measurements at the level of the gating residues it seemed obvious that the ion channel formed by the M3 helices was not permeable to ions from the extracellular side of the cell membrane [29]. However, the structure of the ion selectivity filter formed by the M2 helices was disordered and simple geometry criteria could not be used. On the other hand, the structure clearly contained the glycine and glutamate ligands, which suggested an activated or nearly open receptor.

**Figure 1:**
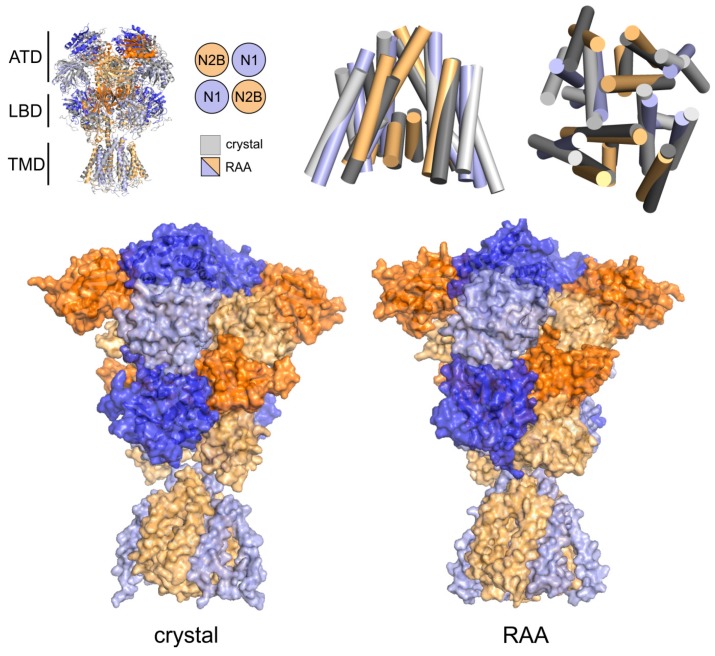
Relaxation of the crystal-based homology model. (**Top**) Summary of the *N*-methyl-d-aspartate (NMDA) receptor architecture showing structure superposition of NMDA receptors (NMDAR) models revealed relatively small differences between the starting crystal structure-based homology model (grey) and the relaxed receptor in the agonist activated receptor (RAA) state shown in blue/orange for GluN1/Glun2B subunits. Superposition of the transmembrane domain led to subtle rearrangement of the outer helices, while the arrangement of central M3 helices was not changed. (**Bottom**) During the relaxation simulation we observed changes in the amino-terminal domain (ATD) and ligand-binding domain (LBD) region, probably as a consequence of the sequence differences between the original crystal structure and the homology model. Overall the receptor kept the crystal structure arrangement and the changes were limited to a slight lifting of the GluN2B NTD domain and rotation around the longitudinal axis of the receptor. The rather flexible linkers connecting the TMD and LBD are not displayed for clarity.

Using the implicit solvent/membrane EEF1/IMM1 MD simulation we relaxed the structure of the homology model including both the glycine and the glutamate ligands, obtaining the agonist activated receptor with the ion channel closed (further referred to as RAA). The RAA state corresponded to a metastable structure connecting the closed to open states of the receptor during its functional transition. A snapshot of the geometry after 20 ns of simulation was used as a representative RAA structure based on the distance of M3 helices of TMD (corresponding to the impermeable channel) while still keeping the symmetrical arrangement of helices. The results are summarized in Figure 1, showing that the largest structural differences were localized to the ATDs, changing the relative orientation of different ATD domains as well as causing reorientation of subdomains within each ATD. The observed rearrangement from the crystal-based homology model towards the relaxed structure included a concerted translation/rotation of the ATDs visualized as a clockwise twist of about 15 degrees around the longitudinal axis and elongation of the whole receptor by lifting of the outer ATD subdomains by about 10 degrees (with respect to the ATD center of mass). The rearrangements at the level of the LBDs and the TMDs were mostly negligible and the ion channel remained in the crystal-like impermeable conformation. The described changes of geometry were probably a result of the sequence differences introduced for the purpose of crystallization.

**Figure 2:**
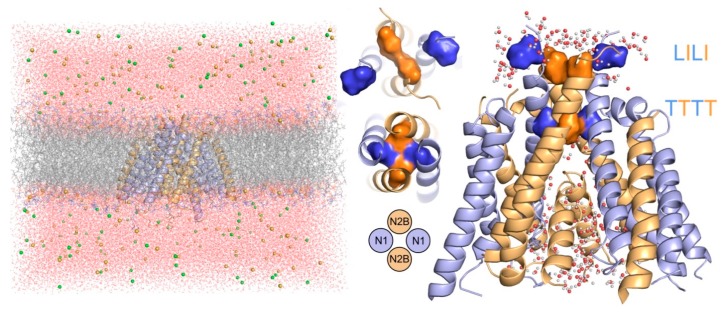
Summary of the explicit solvation all-atom molecular dynamics (MD) simulation of the transmembrane domain of the NMDAR in the RAA state. (**Left**) Snapshot from the 100 ns MD simulation showing the TMD in blue/orange cartoon for the GluN1/GluN2B subunit, anchored in a DOPC membrane (grey/red/blue for carbon/oxygen/nitrogen atoms), and surrounded by water and ions in a simulation box. (**Middle**) Top view of the channel-lining M3 helices. The gating residues of the auxiliary LILI gate formed by Leu657 of GluN1 (blue) and Ile655 of GluN2B (orange) were located above the TTTT gate formed by Thr648 of GluN1 and Thr647 of GluN2B. (**Right**) Zoom of the TMD showing water molecules above and below the gating residues of the auxiliary LILI gate on top of the TTTT gate. During the course of the simulation, there were no water molecules or ions found in the confined space between the LILI and TTTT gates, supporting the impermeability of the channel in the RAA state based on the Thr–Thr distances.

To further assess the permeability of the channel taking into account its dynamics, we performed a 100 ns long explicit solvent/membrane MD simulation of the isolated transmembrane part of the relaxed model. The analysis of the simulation trajectory supported the impermeability of the extracellular hydrophobic funnel between the LILI and TTTT gates for water and ions, while the ion selectivity filter and the cavity accessible from the intracellular space contained a chain of hydrogen-bonded water molecules reaching just below the TTTT gating residues (see Figure 2).

### 3.2. Modeling of the Open State

**Figure 3:**
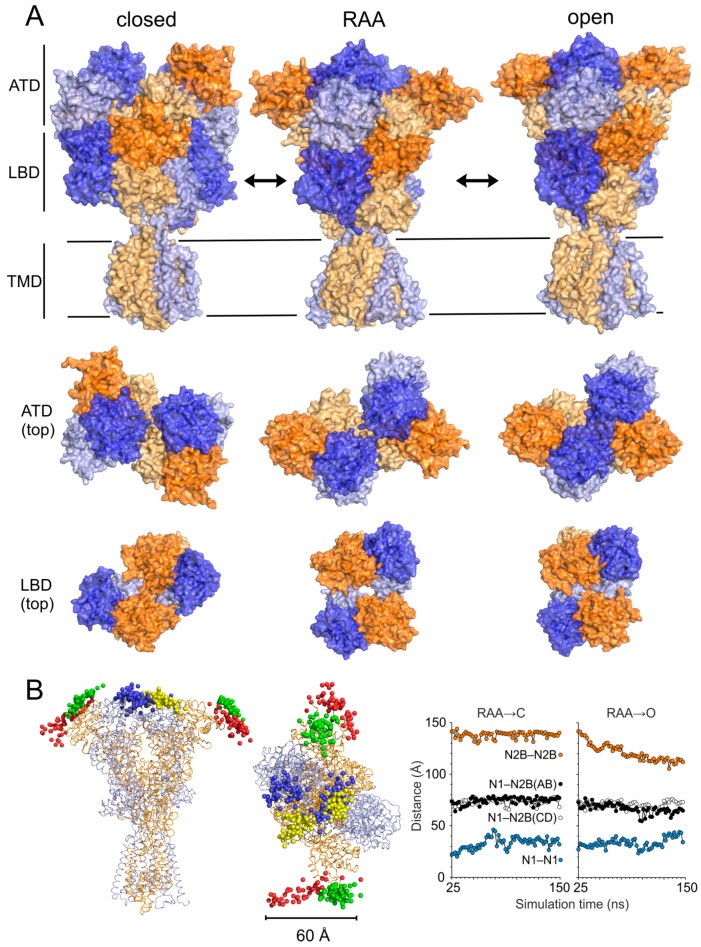
Summary of opening and closing transitions in the NMDAR from MD simulations. (**A**)(**Top**) Side view of the NMDAR showing the structural transition from the closed to the open state of the receptor. The snapshots of geometry from the MD simulation were structurally superimposed using the TMD M3 helices as a reference, keeping the membrane-bound TMD in the same orientation across figures. Different tilt of the outer TMD helices resulting from the iris like movement can be seen. (**Bottom**) The simulation revealed large rearrangement at the level of the ATD and LBD domains during the structural transition between closed and open states. The transition can be described as a rotation of intact ATD and LBD domains from different subunits. The interaction surface of domains changed during the transition as a consequence of the rolling motion. (**B**) (**Left**) Side and top view at the model of NMDAR in the RAA state summarizing the movement of the ATD domains during the opening (green spheres for GluN2B, yellow spheres for GluN1) and closing (red spheres for GluN2B, blue spheres for GluN1) using snapshots of C_α_ atom positions of N-terminal residues. (**Right**) Distances between N-termini of GluN2B–GluN2B ATD (orange), neighboring GluN1–GluN2B ATD (black and white), and GluN1–GluN1 ATD (blue) from the closing MD simulation (left) and opening MD simulation (right). Time 25 ns corresponds to the RAA model. The data show that while the distance between neighboring GluN1/GluN2B domains remained nearly constant during simulations, rotation of GluN2B domains led to shorter distance between termini during the opening simulation. The panel B summarizes data from a single 150 ns opening and single closing 150 ns simulation.

**Figure 4:**
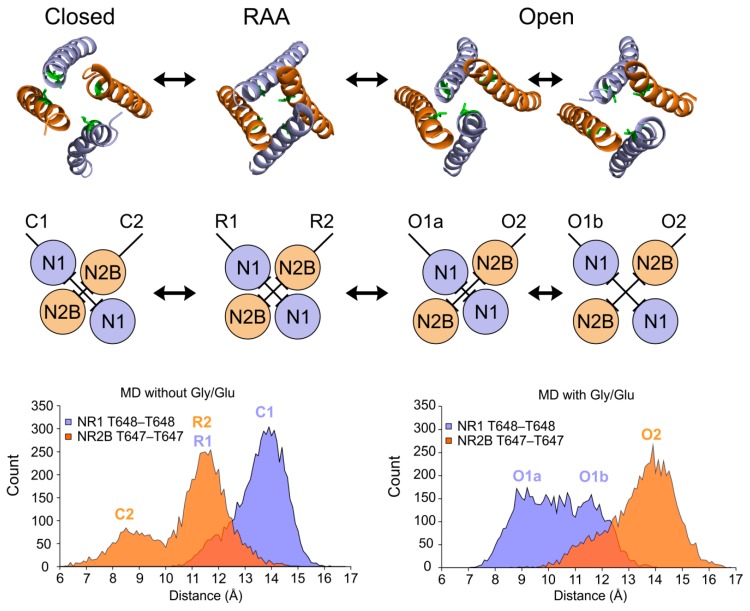
Comparison of the arrangements of inner ion-channel helices (M3 helices of the TMD) between the different functional states of the NMDA receptor. (**Top**) Snapshots of M3 helices from MD simulations of NMDA receptor closing and opening. Threonine residues forming the TTTT gate are shown as green sticks. (**Middle**) In the closed state the MD simulation of the unliganded receptor led to a closer contact of GluN2B M3 helices (orange circles, C2 distance), while GluN1 helices (blue circle) were more separated (C1 distance). Relative M3 helix distances inside the channel pore are indicated by the ├┤ symbols. The RAA state shows the M3 helices in a symmetrical arrangement (R1 and R2 distances), however, this orientation represented a transition between the closed and the open state. Contrary to the unliganded case, the MD simulation of the liganded receptor led to a separation of GluN2B M3 helices as a consequence of the LBD mechanical signal (O2 distance), while the GluN1 M3 helices transiently formed a closer contact (O1a distance). (**Bottom**) Histograms of C_α_–C_α_ distances in Å between the gating threonine residues across the channel extracted from the MD simulation, GluN1–GluN1 (Thr648–Thr648) shown in blue and GluN2B–GluN2B (Thr647–Thr647) in orange. During the opening simulation GluN1 M3 helices oscillated between two maxima of the distance distribution (corresponding to energy minima) with distances between the gating Thr648–Thr648 distances across the channel of around 8.5–11.5 Å. The shorter O1a distance corresponded to the LILI gated state, while the longer O1b distance represents a fully open ion-permeable channel. The histograms summarize data from a single 150 ns opening and single closing 150 ns simulation. The distance codes in the figure were defined as follows: the C? denoted the closed state distances, R? denoted distances in the RAA state, and the O? denoted distances in the open state.

To model the open state of the NMDAR we continued the simulation of the receptor with glycine and glutamate ligands bound for 150 ns. The rearrangement of the ATD and the LBD observed for the RAA state continued. At the ATD level the structure reached an even tighter interaction between the GluN1 subunits as well as between GluN2B and GluN1 (see Figure 3A,B). The rearrangement at the LBD level was smaller but the rotation propagated to the TMD and led to a significant separation of the GluN2B channel-lining M3 helices. In the course of the MD simulation the GluN1 helices first filled the newly accessible space between the M3 helices, blocking the channel. This asymmetry was identified in our previous study and related to the newly described auxiliary LILI gating residues. The combination of vibrations at the level of the M3 helices and the upward pull of GluN1 LBD–TMD linkers due to LBD rotations led to a transiently fully open (conductive) channel. Our simulation protocol led to a distribution of distances at the level of the gating threonine residues between the GluN1 M3 helices with two comparably represented maxima around 8.5 and 11.5 Å (see Figure 4), leading, under the effectively accelerated simulation conditions, to up to 50% open probability, depending on the actual channel diameter necessary for the transport of ions.

### 3.3. Modeling of the Closed State

To complete the description of the NMDAR functional cycle we performed MD simulations of the receptor starting from the RAA state without the glycine and glutamate ligands present. Since the simulations of liganded states nicely reproduced the experimentally available data, this simulation was expected to provide good approximation of the closed state of the receptor. During the simulation we observed significant large-scale rearrangement of the NMDAR domains (see Figure 3A,B). Closing of the receptor in the unliganded state led, at the ATD level, to a larger separation of the GluN1 domains, while the lower lobes of the GluN2B domains were in closer contact across the receptor axis compared to the RAA (as well as the crystal and open receptor) structures. The LBD of GluN1 rearranged with respect to GluN2B and the two GluN2B domains formed a closer contact similarly to the ATD level. The magnitude of the clockwise rotation of ATD and LBD domains reached about 45 degrees around the longitudinal axis of the receptor. Due to the loss of mechanical signal from the GluN2B LBD, the GluN2B M3 helices of the TMD, which were distant in the RAA and the open state, formed a tight contact and served as the dominant channel gate. The GluN1 helices, including the LILI gate-forming residues, were more separated. Both changes lead to an asymmetrical arrangement of the M3 helices, opposite to the previously mentioned activated states. Contrary to the active functional states where the changes at the TMD level were mostly related to the movement of the M3 helices, the loss of mechanical coupling between the LBD and the TMD in the closed state also led to a reverse iris-like movement of the remaining TMD helices. The symmetrical TMD M3 helix arrangement found in the available experimental structures probably represented an averaged or transition structure connecting the closed and open channel arrangements.

## 4. Discussion

Recent all-atom simulations describing the NMDAR as well as solvent and membrane components at the atomic level are reaching millisecond simulation time scales. There is a common and often valid expectation that the classical all-atom MD simulation samples the dynamics of a system on a faster, accelerated time scale. However, under specific conditions, longer simulation times are necessary to describe dynamics of a system corresponding to shorter real times. It is rather difficult to find a common relation of the simulation time scale to the real time, which would in fact mostly depend on the available energy and efficiency of conformational sampling within the system. For a large protein like an NMDAR, which performs large conformational changes, we can also expect significant influence of the viscosity of water environment surrounding the protein. The implicit solvent/membrane EEF1/IMM1 simulation allows effectively for a higher sampling rate compared to all-atom simulation including the solvent and membrane atoms, where the large reorientation of domains can be significantly slower due to necessary rearrangement of the vast number of randomly colliding solvent molecules. It was reported recently [59] that the binding of glutamate and glycine to the LBDs occurs in a guided and random diffusion manner, respectively, and the glutamate ligand can adopt different binding modes within the binding cavity. Due to the faster dynamics of the whole system resulting from using an implicit solvation model, the glycine and glutamate ligands showed relatively low residence times (tens of ns) within the binding pockets of the ligand-binding domains.

### 4.1. Mechanism of NMDAR Ion Channel Opening and Closing

In our simulations we observed a surprisingly large rearrangement of the NMDA receptor during the transition between the closed and open states. The change in the relative orientation of the ligand-binding and the N-terminal domains caused by a perturbation of the electrostatic potential of the GluN2B LBD after binding or unbinding of the negatively charged glutamate ligand was transferred through the linker regions to the membrane-anchored assembly of transmembrane helices. The torque induced by the rearrangement of the extracellular domains was then transformed to an iris-like movement of the central, pore-lining M3 helices. During the simulations without distance restraints, as well as the initial optimization of distance restraint parameters for glycine and glutamate ligands, we observed a fast structural response to the binding and unbinding of ligands at the simulation time scale of nanoseconds. The complete transition between open and closed states in the following production simulations occurred within about 50 ns after the unbinding or binding of ligands to the initial structure. However, as mentioned above, this time scale does not directly relate to the experimentally observed NMDA receptor opening and closing kinetics.

**Figure 5:**
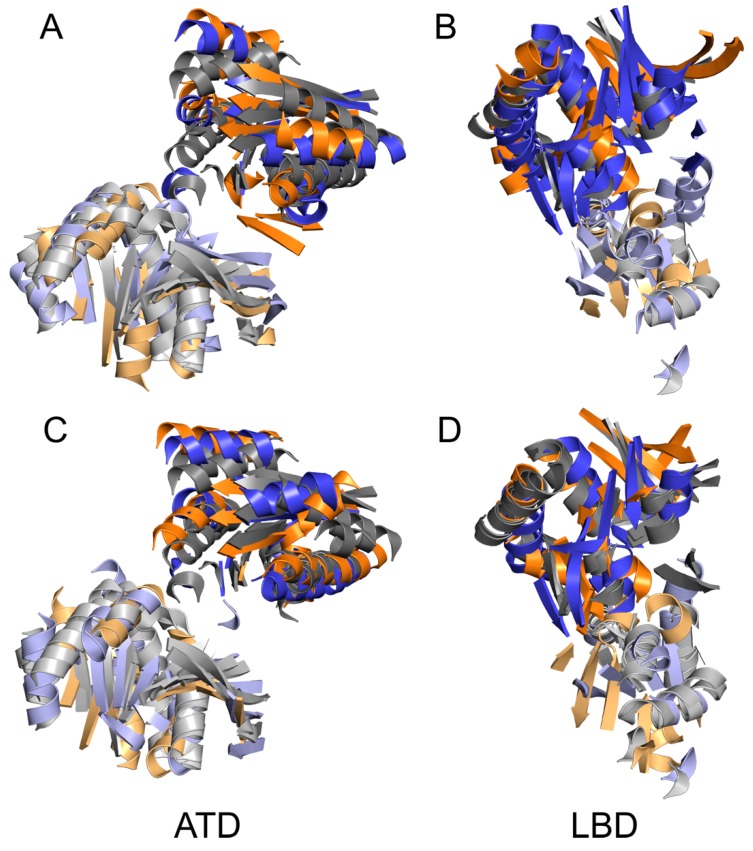
Structural superposition of GluN1 and GluN2B ATD and LBD domains in different functional states of the NMDA receptor. The figure shows structural similarity of GluN1 and GluN2B domains across functional states of the NMDA receptor. For each domain (ATD—left or LBD—right) the closed (**A**,**B**) and open (**C**,**D**) conformations of the GluN1 (blue) and GluN2B (orange) subunits were compared to the crystal structure based homology model of GluN1 and GluN2B subunits (grey). The structural differences between GluN1 and GluN2 subunits were in general comparable to differences observed during the closed to open state structural transition. As expected, the largest difference could be seen for the GluN2B LBD in response to glutamate binding. However, this difference could not fully explain the overall large rearrangement necessary for opening and closing of the receptor.

Concerning the opening and closing mechanism, there are two notable observations. Although the observed rearrangements during the course of the structural transition between the closed and open states were unexpectedly large (see Figure 3), the conformations of isolated ATD and LBD domains showed only limited changes. The structural differences between GluN1 and GluN2 subunits were in general comparable to the differences observed during the closed to open state structural transition (see Figure 5). As expected, the largest difference could be seen for GluN2B LBD in response to the glutamate binding and unbinding. Conformational changes indicating rotational motions of LBD were observed when all-atom explicit solvation MD simulation was used, however, despite 300 ns the timescale was not sufficient to observe complete gating event [37]. Our observation is also in agreement with the “rolling motion” described in the recent study by Esmenjaud et al. [60] based on fitting homology models to experimental cryoEM density. The reported C_α_–C_α_ distances between GluN2B Leu795 and GluN1 Glu698 (7.7 Å) or Arg673 (10.3 Å) compare well with the average distances of 7.9 ± 0.9 Å and 9.9 ± 0.3 Å, respectively, measured from the second half of the MD simulation of the liganded GluN1/GluN2B receptor.

**Figure 6:**
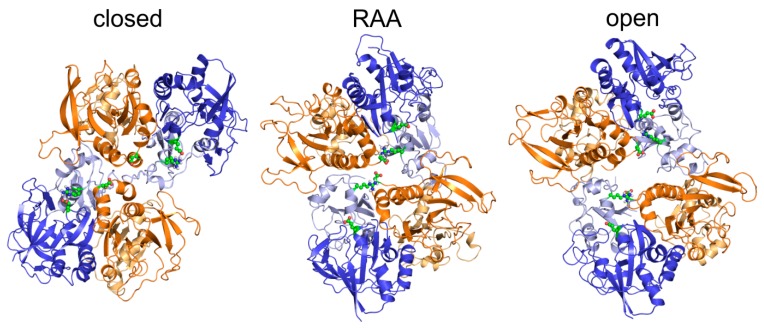
Summary of key LBD residues involved in the NMDAR structural transition between closed and open states. In the open state (and partially also in the RAA state) the Arg695 residue of the lower GluN1 LBD lobe (light blue) formed a double salt bridge with the Asp786 of the upper GluN2B LBD lobe (orange) and the Glu522 residue of the upper GluN1 LBD lobe (blue). In the closed state, however, the GluN1 Arg695 residue interacted only within the subunit with the Glu522 residue of the upper GluN1 LBD lobe (blue). The observed changes in the Asp–Arg–Glu salt bridges were related to the binding and unbinding of the negatively charged glutamate ligand to the GluN2B LBD binding cavity.

Our MD-based approach augmented that study by suggesting a step-by-step description of the experimentally unavailable transitions between the functional states of the NMDAR at an atomic resolution. From the MD simulation data we could extract observations of specific residues changing their binding partners during the transition (see Figure 6). In the open state (and partially also in the RAA state) the Arg695 residue of the lower GluN1 LBD lobe formed a double salt bridge with Asp786 of the upper GluN2B LBD lobe and the Glu522 residue of the upper GluN1 LBD lobe. In the closed state, however, the GluN1 Arg695 residue interacted only within the subunit with the Glu522 residue of the upper GluN1 LBD lobe. The observed changes in the Asp–Arg–Glu salt bridges were a consequence of the binding and unbinding of the negatively charged glutamate ligand to the GluN2B LBD binding cavity.

**Figure 7:**
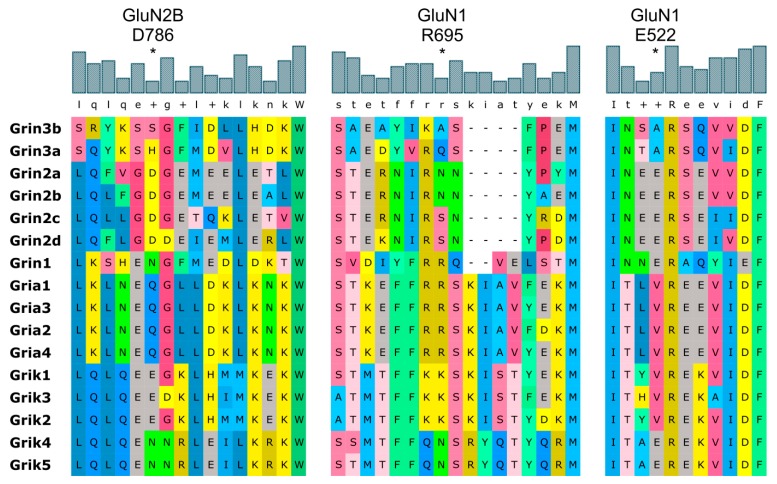
Sequence alignment and conservation of rat iGluRs, showing residues at key LBD interfaces identified from the opening and closing MD simulation of the rat GluN1/GluN2B NMDA receptor. The residues of the Asp–Arg–Glu motif as well as residues in its vicinity showed subunit-specific distribution. The GluN2x subunits can be distinguished by presence of a specific GDG sequence followed by a stretch of negatively charged amino acids.

All three residues involved in the identified salt bridges showed distinct distributions in groups of iGluR subunits forming specific clusters of amino acids (for the summary see Figure 7 and columns 1081 and 877 for GluN1 Arg695 and Glu522 residues in the supplementary sequence alignment uniprot-grin1 fasta file; the GluN2B Asp786 corresponds to the column 1026 in the supplementary sequence alignment uniprot-grin2b fasta file). For example, the GluN2 subunits carried the Asp residue in the sequence corresponding to position 786 of GluN2B, while the remaining AMPA and Kainate receptors contain mostly polar residues instead. The residues were also highly conserved within a subunit across vertebrates. A single case of sequence difference for vertebrates from Asp786 residue was found for the fish Gasterosteus in the UniProt database for the GluN2B *Grin2b* gene, the remaining differences were found in insects and nematodes. The only GluN1 Arg695 and Glu522 sequence differences were found for the Stylophora coral. Especially around the GluN2B GDG motif there are no known mutations reported for the (human) *GRIN2B* gene. This could suggest a functionally important protein region. Interestingly, the GDG protein sequence originates from different exons and the terminal glycine codon is formed after RNA splicing. According to a BLASTP [61] search for the LQLF**GDG**EMEEL sequence of the GluN2B, the GDG motif is nearly 100% conserved (in 273 of 275 hits), with the exception of two uncharacterized fish proteins carrying a Gly codon deletion variant GD-. For more details see columns 1394 to 1396 of the sequence alignment in the supplementary BLASTP_LQLFGDGEMEEL fasta file. There are genomic variants reported in the coding sequence of GluN1 Arg695 and Glu522 residues, however, their functional consequences are currently not known.

### 4.2. Common and Different Structural Features Derived from the Three Independent Simulations

The three independent simulations for the liganded, as well as the unliganded NMDA receptor, provided qualitatively identical results with respect to its global conformational states. The local conformations of the domains and timescales of the observed transitions differed, however. The analysis of simulation data was based on the relative location of the selected residues of the channel-lining M3 TMD helices. All three liganded simulations resulted in the open state with the ion channel oscillating between permeable and impermeable distances of the TTTT gating residues, supporting the suggested opening mechanism, but the local differences between simulations do not allow more detailed description of existing functional sub-states. Due to the large rotation and rearrangements during the closing simulations, the situation is even more complex here. However, in all unliganded closing simulations we consistently observed a significant rotation of the extracellular part with respect to the TMD in a range between 28 and 45 degrees, occurring between 20 and 50 nanoseconds from the beginning of the simulation. Further, in all the closing simulations the ion channel adopted the non-symmetrical arrangement of the M3 helices with the channel remaining impermeable throughout the simulation. Probably as a result of the implicit membrane and solvation protocol we observed a different level of bending of the extracellular domains with respect to the TMD, which further complicated the analysis of the combined simulation and suggested that a significantly longer simulation time would be necessary to describe the closing with sufficient detail for a reliable description of the sub-states.

For a different type of analysis of the structural transition of the NMDA receptor we performed a Markov state model analysis of the simulation data. The Markov states were identified based on the RMSD metric for all C_α_ atoms in order to perform the analysis without an *a priori* selection of “important” residues. The trajectories were clustered according to the RMSD parameter and six states were created using a 0.1 ns lag-time (based on the implied timescale plot (Figure S1). This choice of metric was expected to capture the global conformational changes well, while it might have been less sensitive for the detection of changes in relatively small, yet functionally relevant, regions of the receptor. MSMs were annotated using a structural superposition of the M3 helices of the six obtained MSMs with the initial NMDAR homology model, the representative snapshots of the closed state, RAA, and the open state of the receptor (see Figure S2). As can be seen in Figure S1, there were two Markov states with low equilibrium probability. State 0 corresponded to the initial crystal-like homology model, while State 1 represented the receptor in the RAA state. Both these states showed low free energy values, supporting the observed fast relaxation of the homology model from the crystallization induced structure as well as the proposed metastability of the RAA state. States 2 and 3 corresponded to the closed state with the ion channel impermeable and the channel-lining M3 helices in the asymmetric arrangement. Both states were closed due to the rotation of the extracellular domains with respect to the TMD. They both possessed close contact of the GluN2B gating residues and changes in tilt of the outer TMD helices but differed by the bending of the extracellular domains with respect to the TMD (with the lower populated State 2 less bent). States 4 and 5 represented the overall conformation of the open state but differed at the interface between GluN1 ATD, where State 4 formed a close contact between the domains more frequently. At the level of the M3 TMD helices both the states adopted the asymmetric arrangement with the GluN2B M3 helices separated and the GluN1 helices oscillating between the impermeable and permeable ion channel depending on the state of the auxiliary LILI gate.

Both approaches identified the significant rearrangement of the NMDA receptor during the transition between its closed and open states, however, a significantly longer simulation time as well as experimental validation of identified residues might be necessary for a more detailed assignment of structural and functional determinants of all the electrophysiologically identified functional states and their transitions.

## 5. Conclusions

Based on unbiased all-atom implicit membrane and solvent molecular dynamics simulations we described for the first time structural components of the full functional cycle of the GluN1/GluN2B heteromeric NMDA receptor. The results provided a complete description of molecular mechanisms of NMDA receptor transition from the state with the transmembrane domain and the ion channel in the open configuration to the relaxed unliganded state with the channel closed. We identified distinct structural states of the NMDA receptor and revealed functionally important residues (the Arg695 residue of the lower GluN1 LBD lobe, the Asp786 of the upper GluN2B LBD lobe, and the Glu522 residue of the upper GluN1 LBD lobe). The activated and open states of the receptor were structurally close to the liganded crystal structure, while in the unliganded receptor the extracellular domains performed rearrangement leading to a substantial clockwise rotation of up to 45 degrees around the longitudinal axis of the receptor. Video S1 summarizes this transition between closed and open states of the NMDAR. The newly identified network of the sequentially conserved salt-bridged LBD residues, manifesting sensitivity to the functional state of the receptor, might explain the mechanism of NMDAR structural transitions as well as the functional differences between different glutamatergic receptors. The details of distinct structural states of the NMDA receptor and their transitions can stimulate the development of new compounds (potential drugs) modulating NMDA receptor activity with specificity to a given functional state.

## Supplementary Materials

The following are available online at https://www.mdpi.com/2218-273X/9/10/546/s1, Video S1: Transition between closed and open states of the NMDAR prepared using snapshots from MD simulations of liganded and ligand free receptors. Figures S1 and S2 summarizing results of the Markov state modeling. Supplementary fasta files containing aligned sequences used for the homology modeling and analysis of sequence conservation within *Grin1* and *Grin2b* genes.
