# Supplementary material for: NMDA Receptor Opening and Closing—Transitions of a Molecular Machine Revealed by Molecular Dynamics
Source: Biomolecules. 2019 Sep 28;9(10):546. doi: 10.3390/biom9100546 (PMC6843686; doi:10.3390/biom9100546)
Supplement: Supplementary file 1 [file biomolecules-09-00546-s001.zip › NMDAR_opening_closing_Supplementary_Figures.pdf]

biomolecules

MDPI

Supplementary data for

NMDA receptor opening and closing - transitions of a molecular machine revealed by molecular dynamics.

Jiří Černý [1,2,*], Paulína Božíková [2], Aleš Balík [1], Sérgio M. Marques [3,4] and Ladislav Vyklický [1,*]

1 Institute of Physiology of the Czech Academy of Sciences, 142 20 Prague 4, Czech Republic
2 Institute of Biotechnology of the Czech Academy of Sciences, BIOCEV, Průmyslová 595, 252 50 Vestec, Prague West, Czech Republic
3 Loschmidt Laboratories, Department of Experimental Biology and RECETOX, Masaryk University, Kamenice 5/A13, 625 00 Brno, Czech Republic
4 International Centre for Clinical Research, St. Anne's University Hospital Brno, Pekařská 53, 656 91 Brno, Czech Republic
* Correspondence: jiri.cerny@ibt.cas.cz (J.Č.); Ladislav.Vyklicky@fgu.cas.cz (L.V.)

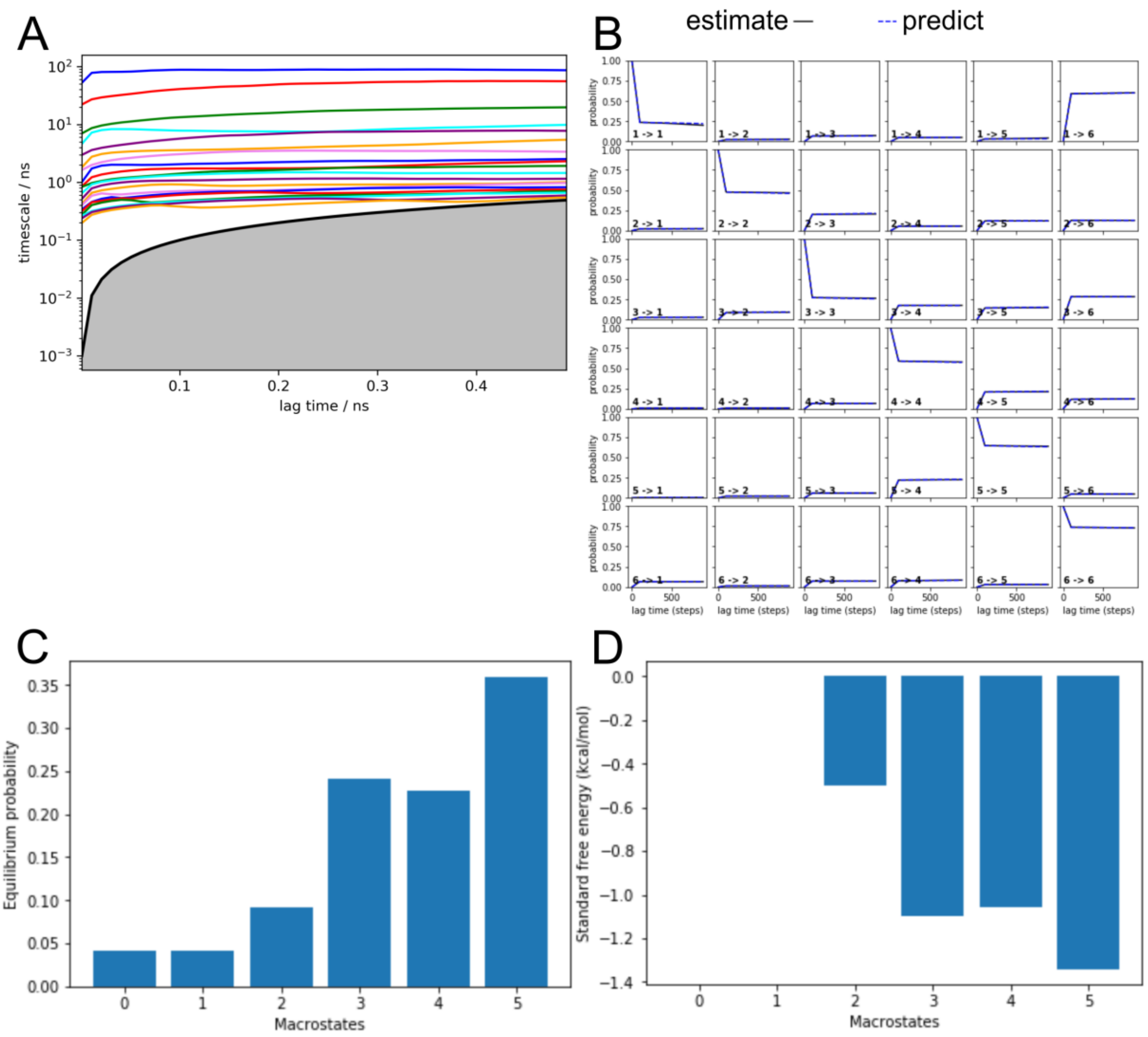

Figure S1. Summary of the Markov state modeling of the combined opening and closing MD simulations of the NMDA receptor. **(A)** The implied timescale plot used for selection of 0.1 ns lag time. **(B)** The results of the Chapman-Kolmogorov test showing the overlap of values supporting a

Markovian behavior of constructed states. **(C)** The equilibrium probability of states. **(D)** The corresponding free energy of states.

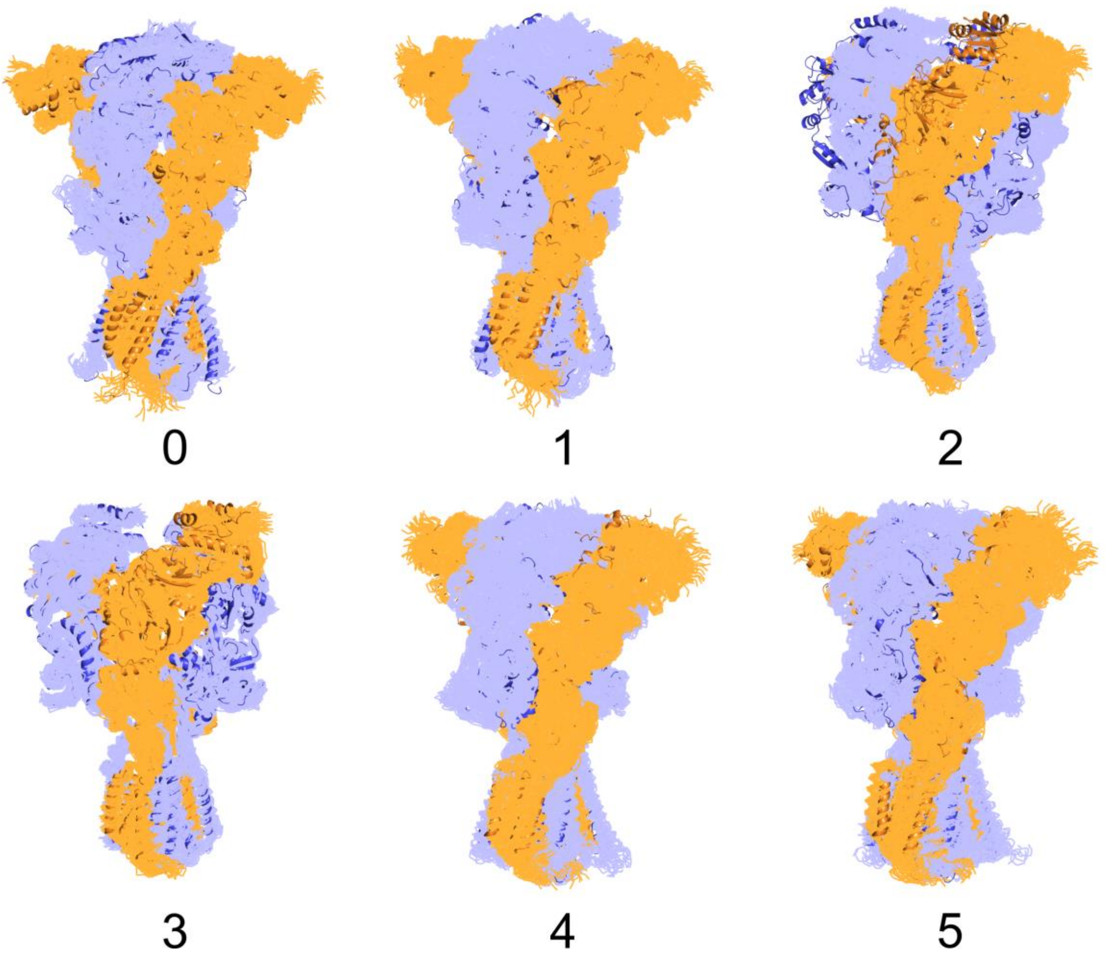

Figure S2. Structure superposition (using the M3 helices) of 50 randomly selected representative structures of the Markov state models from the combined opening and closing MD simulations of the NMDA receptor. The state **0** corresponds to the initial crystal-like homology model, while the state **1** represents the receptor in the RAA state. Both these states show low free energy values (see Supplementary Figure S1), supporting the observed fast relaxation of the homology model from the crystallization induced structure as well as the proposed metastability of the RAA state. The states **2** and **3** correspond to the closed state with the ion channel impermeable and the channel lining M3 helices in the asymmetric arrangement. Both states are closed due to the rotation of the extracellular domains with respect to the TMD. They employ the close contact of the GluN2B gating residues and changes in tilt of the outer TMD helices but differ by the bending of the extracellular domains with respect to the TMD (with the lower populated state 2 less bent). The states **4** and **5** represent the overall conformation of the open state but differ at the interface between GluN1 ATD, where the state 4 forms a close contact between the domains more frequently.

© 2019 by the authors. Submitted for possible open access publication under the terms and conditions of the Creative Commons Attribution (CC BY) license (http://creativecommons.org/licenses/by/4.0/).
